# Potential Mechanisms of Tunneling Nanotube Formation and Their Role in Pathology Spread in Alzheimer’s Disease and Other Proteinopathies

**DOI:** 10.3390/ijms251910797

**Published:** 2024-10-08

**Authors:** Szymon Kotarba, Marta Kozłowska, Małgorzata Scios, Kamil Saramowicz, Julia Barczuk, Zuzanna Granek, Natalia Siwecka, Wojciech Wiese, Michał Golberg, Grzegorz Galita, Grzegorz Sychowski, Ireneusz Majsterek, Wioletta Rozpędek-Kamińska

**Affiliations:** 1 Department of Clinical Chemistry and Biochemistry, Medical University of Lodz, 92-215 Lodz, Poland; szymon.kotarba@stud.umed.lodz.pl (S.K.); marta.kozlowska1@stud.umed.lodz.pl (M.K.); malgorzata.scios@stud.umed.lodz.pl (M.S.); kamil.saramowicz@stud.umed.lodz.pl (K.S.); julia.barczuk@stud.umed.lodz.pl (J.B.); zuzanna.granek@stud.umed.lodz.pl (Z.G.); natalia.siwecka@stud.umed.lodz.pl (N.S.); wojciech.wiese@stud.umed.lodz.pl (W.W.); grzegorz.galita@umed.lodz.pl (G.G.); grzegorz.sychowski@stud.umed.lodz.pl (G.S.); ireneusz.majsterek@umed.lodz.pl (I.M.); 2 Department of Histology and Embryology, Medical University of Lodz, 90-419 Lodz, Poland; michal.golberg@umed.lodz.pl

**Keywords:** Alzheimer’s disease, tunneling nanotubes, dementia, etiopathogenesis, Tau proteins, beta-amyloid, TDP-43, alpha-synuclein

## Abstract

Alzheimer’s disease (AD) is the most common type of dementia worldwide. The etiopathogenesis of this disease remains unknown. Currently, several hypotheses attempt to explain its cause, with the most well-studied being the cholinergic, beta-amyloid (Aβ), and Tau hypotheses. Lately, there has been increasing interest in the role of immunological factors and other proteins such as alpha-synuclein (α-syn) and transactive response DNA-binding protein of 43 kDa (TDP-43). Recent studies emphasize the role of tunneling nanotubes (TNTs) in the spread of pathological proteins within the brains of AD patients. TNTs are small membrane protrusions composed of F-actin that connect non-adjacent cells. Conditions such as pathogen infections, oxidative stress, inflammation, and misfolded protein accumulation lead to the formation of TNTs. These structures have been shown to transport pathological proteins such as Aβ, Tau, α-syn, and TDP-43 between central nervous system (CNS) cells, as confirmed by in vitro studies. Besides their role in spreading pathology, TNTs may also have protective functions. Neurons burdened with α-syn can transfer protein aggregates to glial cells and receive healthy mitochondria, thereby reducing cellular stress associated with α-syn accumulation. Current AD treatments focus on alleviating symptoms, and clinical trials with Aβ-lowering drugs have proven ineffective. Therefore, intensifying research on TNTs could bring scientists closer to a better understanding of AD and the development of effective therapies.

## 1. Introduction

Alzheimer’s disease (AD) is the most common cause of dementia [1]. According to the World Health Organization (WHO), approximately 50 million people worldwide are living with dementia, with AD accounting for 60–70% of cases [2]. It is estimated that by 2050, the prevalence of AD will double in Europe and triple worldwide [3]. AD has a progressive course and is characterized by symptoms such as confusion, irritability, aggression, mood swings, language difficulties, and long-term memory loss. Patients withdraw from family and social life, experiencing a loss of vital functions that leads to death [4,5].

The pathophysiology of AD has still not been fully elucidated. There are several theories attempting to explain the pathophysiology of AD [6]. The cholinergic hypothesis posits that AD symptoms arise from diminished acetylcholine-dependent neurotransmission [6,7]. Another hypothesis is neuroinflammation, emphasizing the role of brain immune mechanisms related to microglia activation [8].

Two of the most important hypotheses regarding the underlying causes of AD are the amyloid-beta (Aβ) hypothesis and the Tau hypothesis. Both proteins are part of the two major pathological lesions in the AD brain: amyloid plaques and neurofibrillary tangles (NFTs), composed of Aβ fragments and hyperphosphorylated Tau, respectively [9,10].

Aβ is the main component of neuritic plaques in AD, and its accumulation has been considered the molecular driver of AD pathogenesis and progression [11,12,13,14]. The most frequently voiced objection to the amyloid hypothesis is that the number of amyloid plaques—fibrous aggregates of Aβ—in the brain does not strongly correlate with the degree of cognitive impairment the patient experienced during life. [15,16,17]. A response to this objection notes that non-fibrillar amyloid aggregates may be more toxic than fibrils. These aggregates include Aβ oligomers consisting of fewer than 50 Aβ molecules and protofibrils including hundreds of molecules [13].

NFTs containing hyperphosphorylated Tau are another hallmark of AD pathology [18], and Tau is a known mediator of Aβ cytotoxicity [19,20]. Tau is a microtubule-binding protein, and Tau phosphorylation at multiple sites controls its binding to microtubules. Under pathological conditions, hyperphosphorylation of Tau protein leads to the dissociation of Tau protein from microtubules and the formation of NFTs. NFTs are abnormal filaments of the hyperphosphorylated Tau protein [21,22,23].

Two other proteins with a significant role in AD pathophysiology are alpha-synuclein (α-syn) and transactive response DNA-binding protein of 43 kDa (TDP-43). α-syn is a major component of Lewy bodies, which are a hallmark of Parkinson’s disease (PD) [24,25,26]. Accumulation of α-syn has also been observed in the brains of AD patients. α-syn interacts directly with Aβ and Tau, promoting their mutual aggregation and worsening cognitive decline [27,28,29,30,31,32]. Recent studies indicate that asymptomatic Aβ plaque accumulation is linked to higher cerebrospinal fluid (CSF) α-syn levels in individuals at risk for sporadic AD and those with autosomal dominant AD mutations. Additionally, α-syn is associated with Tau hyperphosphorylation and the pathological effects of Aβ [33,34,35]. Another significant protein is TDP-43, which is involved in RNA splicing and gene regulation. TDP-43 forms cytoplasmic inclusions that are characteristic of amyotrophic lateral sclerosis (ALS) and some forms of frontotemporal degeneration (FTLD). It is worth noting that TDP-43 inclusions are present in up to 57% of AD cases. In some cases, TDP-43 deposits coexist with NFTs. AD patients with TDP-43 pathology show more severe cognitive impairment compared to those without pathology [36,37,38].

Recently, attention has been brought to the potential role of tunneling nanotubes (TNTs) in the etiopathology of AD and their potential involvement in the specific spreading of the misfolded proteins within the brain [39]. TNTs are thin membranous structures that interconnect physically separated cells [40]. These cellular extensions facilitate the transfer of various cargoes, ranging from small molecules like ions and macromolecules (nucleic acids, proteins, etc.) to entire organelles (vesicles, lysosomes, mitochondria, autophagosomes) between connected cells. By linking multiple cells, TNTs contribute to the formation of functional cellular networks [41,42,43,44]. They are found in various cell types, including neuronal cells, epithelial cells, and nearly all immune cells [45], and are essential for development, tissue homeostasis, and regeneration [46].

TNTs have been suggested to play a significant role in maintaining neuronal networks. They contribute to the transmission of electrical signals, regulate immune responses in the CNS, and may influence brain development [47,48,49]. Furthermore, TNTs have been observed in various disease states, including cancer and neurodegenerative diseases. They may facilitate tumor development, invasion, and metastasis and are involved in transferring pathogenic agents, such as bacteria, viruses, and prions, between cells, thereby aiding the spread of diseases [50,51]. In pathological conditions affecting the CNS, such as AD and PD, TNTs might play a role in the spread of pathological proteins [52,53,54,55]. Since TNTs can facilitate the direct transfer of Tau aggregates from one neuron to another, bypassing the extracellular space, they can thus avoid degradation by microglia and extracellular enzymes. TNTs may help explain the predictable pattern of Tau spread across distant brain regions (Braak staging) [56] and the differing susceptibility of neuronal populations to Tau aggregation and resulting damage (selective neuronal vulnerability). Selective vulnerability of neurons is strongly associated with cellular stress [57], likewise the formation of TNTs. Indeed, the formation of TNTs is particularly notable under oxidative stress conditions. Oxidative stress, known for its critical role in nerve cell dysfunction and death in AD, is extensively linked to the neurotoxic effects of Aβ. Aβ induces oxidative stress, which may initiate TNT formation as a cellular response [58,59]. Therefore, the oxidative stress caused by Aβ in AD may promote the formation of TNTs, contributing to the progression of neurodegenerative processes.

Despite numerous hypotheses, the precise pathomechanism of AD remains elusive [60]. This review aims to explore the mechanisms of TNT formation and their impact on the etiology and progression of AD and to establish connections between TNTs and other concepts related to the disease’s causes.

Over the past 30 years, most efforts to find an effective AD therapy have focused on clearing Aβ from the brain, as the amyloid cascade hypothesis suggested this peptide was the key pathogenic factor in AD. However, Aducanumab and Lecanemab, two anti-Aβ antibodies recently approved by the FDA for AD therapy, demonstrated limited efficacy on cognitive outcomes despite significantly reducing Aβ brain load [11,61,62,63,64]. Current approved treatments for AD, such as acetylcholinesterase inhibitors (AChEIs) and memantine, focus only on managing symptoms by addressing neurotransmitter imbalances [65,66]. The potential for TNTs to alleviate the neuronal burden of α-syn—one of the key pathological proteins implicated in AD—highlights TNTs as a therapeutic avenue in the treatment of AD.

## 2. Pathologic Aggregation and Spreading of Misfolded Aβ and Tau Protein in AD Pathogenesis

There are two fundamental paradigms common among proteopathies—the phenomenon of self-aggregation and intercellular transmission of aberrant proteins. Self-aggregation presupposes that an abnormally folded pathogenic protein in contact with an endogenous protein induces a change in its conformation initiating protein self-assembly, which leads to the formation of larger aggregates. The resulting aggregates such as oligomers act as seeds that can effectively recruit their soluble counterparts to form β-sheet-rich fibrils. It is believed that soluble oligomers are the major culprit of neurotoxicity and pathology propagation in neurodegenerative diseases. A small number of aggregates can also be secreted from the host cell into the extracellular matrix (ECM) and then taken up by the recipient cell. This cell-to-cell transmission leads to pathology dissemination throughout the CNS and is responsible for unique manifestations of each proteinopathy. Interestingly, the spreading of pathology seems to reflect a predictable spatiotemporal pattern of progression through anatomically or physiologically related parts of the human brain [67,68,69,70,71,72,73,74,75].

### 2.1. Ab and Tau Aggregation and Formation of Pathogenic Species

Aβ peptides (monomers) originate from proteolytic processing of transmembrane Amyloid Precursor Protein (APP), abundantly expressed in the synaptic membrane of neurons. APP is synthesized in the endoplasmic reticulum (ER) and then trafficked to the trans-Golgi network to complete maturation and eventually be transported to the plasma membrane [76,77]. Proteolytic cleavage of APP can proceed via two different routes: amyloidogenic and non-amyloidogenic. In the physiological non-amyloidogenic pathway, APP is transported to the cell membrane, where it is cleaved by α-secretase (enzymes of the ADAM proteases family such as ADAM9, ADAM10, and ADAM17) to generate the neuroprotective soluble fragment sAPPα [78]. Dissociation of sAPPα leaves an 83-amino acid C-terminal fragment of APP (C83) that precludes Aβ formation. In contrast, when APP is cleaved by β-secretase (encoded by β-APP-cleaving enzyme-1 (BACE1)), it produces a 16 amino acid longer 99-amino acid C-terminal fragment of APP (C99), which after subsequent cleavage by γ-secretase releases Aβ40/42 amyloidogenic species. After dissociation, Aβ can be released directly into the extracellular space, bounded with lipid raft structures, degraded by proteolytic enzymes (such as neprilysin, insulin-degrading enzyme or endothelin-converting enzyme), and internalized by receptor-mediated endocytosis [79,80,81,82]. A substantial portion of Aβ can also be produced in the membranes of the trans-Golgi network and early/late endosomes, as their acidic environment provides the proper conditions for optimal BACE1 enzymatic activity [83,84].

APP-derived Aβ monomers can form higher-order assemblies including soluble oligomers and protofibrils and insoluble β-sheets-rich fibrils. The aggregation trigger is not entirely known, however, the intrinsically disordered nature of Aβ and interactions with environmental elements such as lipid membranes and ECM proteins as well as post-translational modifications like glycation or N-terminal truncation increase the propensity of Aβ to form aggregates [85,86,87,88]. The aggregation process is believed to begin with primary nucleation, which generates a seed (nucleus)—the smallest stable oligomer capable of promoting further aggregation. This is followed by the elongation, characterized by the addition of monomers to the nuclei, which successively leads to the formation of larger aggregates [88]. The resulting aggregates can constitute new nuclei, further promoting Aβ aggregation (a process known as secondary nucleation) [89]. It has been demonstrated that cross-linked Aβ dimers may represent the earliest Aβ assemblies with neurotoxic and synaptotoxic properties [90,91]. Larger soluble oligomers are considered the most detrimental Aβ species, that significantly alter the neuronal membrane permeability via its excessive perforation and creation of ion channels, induction of a signal of cell apoptosis, and an extensive neuroinflammatory response across CNS [92,93]. Furthermore, although extracellular Aβ accumulation in neuritic plaques has classically been considered a hallmark of AD pathology, recent studies suggest that intracellular Aβ oligomers may represent a more relevant species in terms of pathology dissemination [94].

Tau monomers in the CNS comprise a mixture of six isoforms generated by alternative splicing of exons 2, 3, and 10 of the *MAPT* (Microtubule Associated Protein Tau) gene. These isoforms can be subdivided into two major categories, known as 3R Tau and 4R Tau, based on whether they contain three or four tubulin-binding repeats on the C-terminus. In AD, aggregates contain both 4R and 3R Tau isoforms [95,96,97]. Physiologically, all isoform types are abundantly expressed in neuronal axons in equal proportions, where they exhibit a plethora of functions such as stabilization and spacing of microtubules, interplay with cellular transcriptome and regulation of axonal transport, neuronal plasticity, and even brain insulin signaling [98,99,100]. Importantly, phosphorylation, among other post-translational modifications, can alter the local tertiary structure of Tau. Notwithstanding the fact that Tau is intrinsically disordered, the phosphorylation process can either stabilize or occlude its dynamic structural changes. In AD, this equilibrium is shifted in favor of hyperphosphorylation, which leads to the dissociation of intrinsically disordered Tau into the cytoplasm, where it begins the process of self-assembly [101]. Tau hyperphosphorylation is the product of deregulated Ser/Thr kinases, such as CDK-5 and GSK-3β [102]. Aβ accelerates the hyperphosphorylation of Tau by mediating the activation of CDK-5 and GSK-3β [102,103]. GSK-3β, which is inextricably related to Aβ, is an important factor in the phosphorylation of Tau, and it aggravates Tau-induced neurotoxicity [104,105]. Accordingly, it is believed that aggregated Aβ induces Tau hyperphosphorylation by enhancing the activity of GSK-3β and CDK-5. However, Tau hyperphosphorylation is not utterly dependent on Aβ-related mechanisms and may result from pro-inflammatory activation of microglia, disturbances in cholesterol metabolism, and the endosomal-lysosomal system [106,107]. In addition to phosphorylation, other post-translational mechanisms, such as acetylation, ubiquitination, and N-terminal truncation, are involved in promoting Tau aggregation and toxicity [108,109,110]. Interestingly, some modifications have been shown to be disease-specific, allowing for differentiation across tauopathies [111]. The process of Tau aggregation proceeds similarly to Aβ through primary nucleation, elongation, and secondary nucleation, as described above [112]. Tau dimers have been demonstrated to be the earliest culprit for Tau neurotoxicity and propagation in vitro and in vivo and may represent the initial nuclei that subsequently self-assemble to form larger oligomeric species [91,113]. Notwithstanding the fact that NFTs constitute a pathological hallmark of AD, there is a growing consensus on the primacy of small soluble Tau oligomers in the induction of neuronal dysfunction and the propagation of Tau pathology [114].

Interestingly, Tau and Aβ act synergistically to drive their neurotoxicity and dissemination. The prevailing hypothesis states that Aβ is upstream of Tau in AD pathogenesis and a direct trigger of pathological aggregation and spreading of Tau. In fact, even small intracellular Aβ oligomers can directly induce Tau hyperphosphorylation [115], while extracellular Aβ species can act as a template that accelerates aggregation and facilitates propagation of phosphorylated Tau (pTau) to distant brain regions, significantly enhancing neuronal loss [116,117]. On the other hand, pTau can potentiate Aβ production, facilitate Aβ deposition, and promote toxicity of Aβ oligomers. Therefore, it has been hypothesized that Tau pathology may initiate a vicious cycle in AD pathogenesis, in which phosphorylated Tau increases Aβ production, and Aβ oligomers, in turn, promote further accumulation of toxic Tau species [80,118,119]. This phenomenon of direct reciprocal interaction between Aβ and Tau is referred to as cross-seeding, and its occurrence along functional connections has been found to promote the onset and acceleration of Tau propagation [120,121]. The spread of Tau directly corresponds with the clinical manifestations of AD [122]. suggesting that pTau could potentially be a causal factor in the pathogenesis of AD, but Aβ may be a prerequisite for the initial seeding of Tau, representing the “sine qua non” of Tau neurotoxicity.

### 2.2. Cellular Mechanisms of Pathology Transmission

To fully capture the phenomenon of Aβ and Tau propagation it is crucial to elucidate the cellular mechanisms underlying the flow of misfolded proteins between neurons and glial components of the neurodegenerative microenvironment. AD patients exhibit increased concentrations of Tau and, limited to the initial phase, of Aβ oligomers in the CSF, which indicates that pathogenic aggregates are released from cells into the extracellular space (ECS) [123,124,125]. This release can occur through passive mechanisms, such as leakage of intracellular contents from dying cells, as well as through active processes involving a wide array of secretory pathways. It is well established that Aβ and Tau spread through synaptic contacts, and the increased presynaptic neuronal activity can stimulate the release and trans-synaptic transfer of the pathogenic proteins [126,127,128,129].

This indicates that Aβ and Tau may be secreted through exosomes or ectosomes, two types of extracellular vesicles associated with presynaptic activity and neuronal depolarization [130,131,132,133,134,135]. Indeed, both exosomes and ectosomes have been found to mediate the secretion of toxic Ab oligomers and pTau species [130,136,137]. Furthermore, although neuronal activity enhances APP processing, alterations in extracellular Aβ levels are predominantly modulated by processes associated with synaptic vesicle release [138]. Hyperphosphorylated Tau species may also be secreted in vesicle-free form by direct translocation across the plasma membrane [139].

Once secreted into the ECS, Aβ and Tau seeds can be taken up by neighboring cells, where they undergo prion-like templating, leading to the formation of new aggregates and propagation of pathology. Aβ oligomers are internalized primarily by clathrin-dependent receptor-mediated endocytosis mediated by ApoE-binding receptors such as low-density lipoprotein receptor-related protein 1 (LRP1) [140,141]. However, Aβ-induced internalization and neurotoxicity can also occur independently of clathrin in the RhoA-regulated dynamin-dependent pathway [142]. Another crucial mechanism of Aβ oligomers internalization is through binding to heparan sulfate proteoglycans (HSPGs), which promote the rearrangements of the plasma membrane and Aβ internalization via micropinocytosis [143]. Tau oligomers are primarily internalized by bulk endocytosis at the somatodendritic compartment, or the axon terminals, and can be transported bidirectionally (anterogradely and retrogradely) within recipient neurons through the endolysosomal system [144]. However, HSPG-dependent macropinocytosis also appears to play a pivotal role in the uptake of pathological Tau species [145]. Upon internalization of Aβ and Tau seeds from the ECS into endosomes, they must find a way to egress from endosomal vesicles in order to interact with monomers and drive self-propagation. Inside the endolysosomal system, Aβ and Tau oligomers can be degraded by lysosomal proteolytic enzymes but most of them escape degradation. This occurs through permeabilization of the endosomal/lysosomal membrane, which readily culminates in enhanced cytosolic Aβ and Tau aggregation and neurotoxicity [146,147].

Aβ and Tau can be engulfed by supporting cells of the neuronal microenvironment including astrocytes and microglia, which may alleviate the proteinaceous burden by degradation of Aβ and Tau aggregates but also induce neuroinflammation and increase pathology spreading [148,149]. Whether astrocytes and microglia are promoters or rescuers of pathology spreading in AD remains debatable. Nevertheless, there is growing evidence that microglial and astrocytic surveillance of the neuronal environment effectively promotes the degradation of extracellular Aβ and Tau aggregates, limiting their propagation by preventing neuronal uptake [150,151,152]. Thus, it is plausible that direct cell-to-cell transmission, bypassing any contact with the ECS, may be a more efficient mechanism for the spread of pathology. To date, the only known route for the direct transfer of misfolded proteins between neurons and glial cells occurs via TNTs.

## 3. TNT Structure, Biogenesis, and Significance in Health and Disease

### 3.1. Potential Mechanisms of TNT Formation

Cells can communicate through numerous mechanisms such as direct interactions, extracellular vesicles, exosomes, and gap junctions [153,154]. Moreover, nanotubular structures forming a complex network are also believed to take part in cell-to-cell communication and transport [155,156,157]. TNTs are actin filaments (F-actin) based membranous tubular structures that facilitate intercellular communication by connecting two cells, spanning up to 300 micrometers [39]. Cryo-electron microscopy observations have revealed that single TNTs are often composed of a bundle of individual TNTs (iTNTs), where each iTNT is enveloped by a plasma membrane and interconnected to others through bridging threads that contain N-cadherin [158]. Through TNTs, cells can transmit electrical signals, chemical compounds, proteins, genetic material, and even entire organelles [49,154,159]. Recently, a proposal has been made to classify TNTs based on their diameter. According to this classification, “thin” TNTs, with a diameter ranging from 20 to 700 nanometers, facilitate the exchange of smaller cargo such as secondary messengers, small peptides, and molecules below 1.2 kDa. On the other hand, “thick” TNTs, with a diameter exceeding 700 nanometers, may contain microtubules stabilizing them and enabling the transport of larger cargo such as organelles or viruses [160,161].

**Figure 1:**
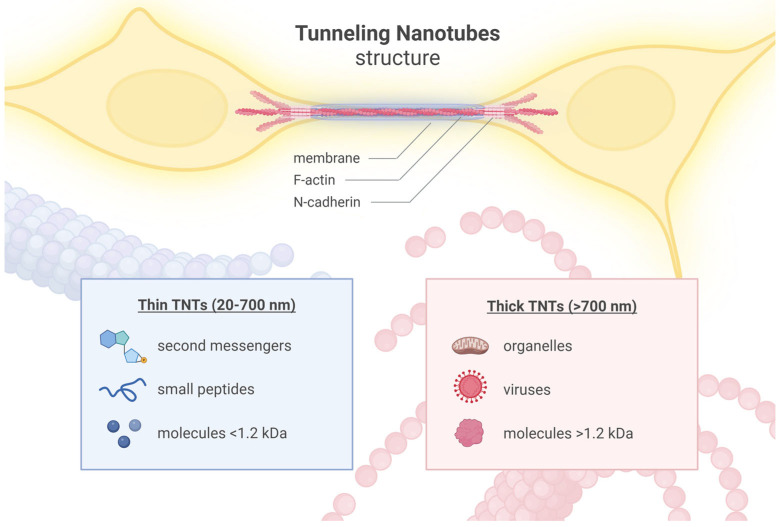
Tunneling nanotubes (TNTs) are tubular, membranous structures that contain F-actin. These structures often consist of bundles of individuals (iTNTs), where each iTNT is encased by a plasma membrane and interconnected with others through bridging threads containing N-cadherin. TNTs are classified into two categories based on their diameter: “thin” TNTs and “thick” TNTs. “Thin” TNTs, ranging from 20 to 700 nanometers in diameter, primarily facilitate the exchange of smaller cargo, such as secondary messengers, small peptides, and molecules with a molecular weight below 1.2 kDa. Conversely, “thick” TNTs, which exceed 700 nanometers in diameter, are capable of transporting larger cargo, including organelles, viruses, and molecules larger than 1.2 kDa.

Misfolded proteins in AD, such as Aβ peptides (4 kDa) and Tau proteins (50–70 kDa), are likely transported through “thick” TNTs, as their sizes exceed the 1.2 kDa weight limit for transport through “thin” TNTs [162,163] (Figure 1) (Table 1).

Importantly, TNTs identification is often challenging, which stems from their fragile nature and susceptibility to numerous stimuli such as light, oxidative stress, and chemical fixation. Additionally, no specific or universal marker for TNTs has been reported thus far [164]. Since 2004, when TNTs were first described [153], numerous studies investigated the potential mechanisms of formation of these structures. However, the results are ambiguous and do not suggest a universal mechanism of TNTs biogenesis [165].

F-actin is suggested to play a major role in regulating the formation of TNTs [166]. The use of Cytochalasin B (CytoB), an F-actin inhibitor, has been shown to reduce the number of junctions between cells while exhibiting a minimal impact on the pre-existing TNTs’ stability [166]. Another compound, tolytoxin, a cyanobacterial macrolide that induces fragmentation of F-actin, has been able to decrease the number of TNT connections [167]. Altogether, these results provide compelling evidence for the involvement of F-actin in the formation of TNTs. While F-actin depolymerization reduces TNT formation, the splicing isoform of microtubule-associated monooxygenase, calponin, and LIM domain containing 2 (MICAL2PV), can also act as TNT suppressors [168].

It is important to acknowledge that the number of TNT connections is not constant and may increase under unfavorable conditions, such as pathogen infections, oxidative stress [169], inflammation [55], misfolded protein accumulation, and pathogenic Aβ aggregates [52,55]. Reactive oxygen species (ROS), including mitochondrial ROS (mtROS), are known to increase the number of TNTs. Excessively generated by oxidative stress in glial cells, ROS/mtROS have been shown to cause damage to mitochondria and induce the activation of the AKT/PI3K/mTOR pathway, leading to the formation of TNTs. It is suggested that in these glial cells, TNTs mediate the transport of healthy mitochondria from healthy donors to disturbed cells to prevent apoptosis caused by disrupted ATP production [170].

The unconventional actin molecular motor protein, Myosin-X (Myo10) is considered a key regulator in the formation of TNTs, enabling neuron-to-neuron transport and contributing to the spread of pathological proteins in neurodegenerative diseases. Additionally, p53 activation and subsequent EGFR upregulation constitute another mechanism through which cellular stress might lead to TNT formation, as confirmed in stress-induced podocytes [171,172,173]. Nevertheless, TNTs are also observed in p53-deficient cells, suggesting that the process of TNT formation is multifaceted and related to a general imbalance in cellular homeostasis rather than the induction of a single pathway [174].

Research on the regulation of TNT formation does not provide a complete picture; there is insufficient data, and the results are inconclusive. Continued research using models or in vivo studies, or the development of improved imaging methods for TNTs, could yield more answers in this area, which could also shed light on the transport of misfolded proteins in AD (Figure 1).

### 3.2. TNTs—Role in Physiology and Pathology

TNTs are suggested to play a significant role in cellular physiology [175,176,177,178]. Through these dynamic structures, cells can exchange information, enabling them to respond more rapidly to stimuli and to adapt faster [175]. Via TNTs, damaged cells can acquire organelles, such as mitochondria, from healthy cells, increasing their chances of survival. TNTs also transport calcium ions involved in regulating neuronal proliferation, migration, and differentiation [179,180]. Moreover, by facilitating signal transmission and contributing to an efficient cellular immune response, TNTs are suggested to be involved in neurogenesis [172,181,182].

In addition to their role in the physiological processes, TNTs may also mediate the development of diseases and the spread of infections [183,184,185,186]. It has been shown that viruses, such as Human Immunodeficiency Virus (HIV), herpesviruses, influenza viruses, and Severe Acute Respiratory Syndrome Coronavirus 2 (SARS-CoV-2), may use TNTs originating from infected cells, as a conduit to infect neighboring healthy cells while also reducing the risk of recognition by the immune system outside the plasma membrane [161,185,187,188]. Moreover, TNTs are thought to play a role in the formation and spread of multiple cancers such as bladder, prostate, pancreatic, and breast cancer, as well as numerous types of leukemia [158,160,189,190,191,192]. In glioblastoma (GBM), increased TNT formation has been observed in cells treated with radiation therapy and temozolomide, which are commonly used in this cancer’s treatment. These connections may facilitate the transfer of tumor-derived mitochondria into nearby healthy cells, enabling their adaptation to altered tumor-related metabolism and hypoxic conditions. Additionally, TNT formation has been detected in resected GBM tumors, potentially confirming the presence of the proposed mechanism in vivo [193].

It is suggested that the development of neurodegenerative diseases is also associated with TNTs. Tau protein, prions, α-syn, and Aβ may be transported between cells via TNTs, promoting protein misfolding and aggregation, contributing to the progression of these diseases [153,194,195]. In Huntington’s disease, TNT-like protrusions have been shown to facilitate the intercellular transfer of polyglutamine-expanded huntingtin (mHTT), contributing to the spread of mHTT aggregates associated with disease progression. Rhes (*RASD2*), a thyroid hormone-induced gene that modulates motor activity in the striatum, facilitates the movement and transport of mHTT through membrane TNT-like protrusions between cultured neurons and also promotes the formation of TNT-like protrusions in medium spiny neurons (MSNs) of the striatum [196,197]. Additionally, TNTs may play a significant role in the progression of PD. Neuronal precursors derived from human induced pluripotent stem cells (iPSCs) have demonstrated the ability to form TNTs facilitating the cell-to-cell transport of α-syn [198]. The presence of α-syn aggregates promotes the formation of TNTs. α-syn aggregates are primarily transferred from neurons to microglial cells, which may help alleviate the buildup of these aggregates. However, mitochondria from healthy microglial cells are transported to α-syn-burdened neurons, probably to rescue the affected cells [199].

TDP-43 and superoxide dismutase (SOD-1) are proteins associated with ALS, among other conditions. Recently, it has been discovered that these proteins can be transported via TNTs, which may influence the progression of the disease [200,201].

## 4. TNTs’ Role in AD

TNTs have been investigated in the context of various pathologies, including neurodegenerative diseases. Numerous studies have been conducted on both in vitro and in vivo models, to elucidate mechanisms of TNT formation, structure, and roles in AD. Multiple amyloidogenic proteins such as Aβ, Tau, prions, and α-syn have been suggested to utilize TNTs as a route for spreading from one cell to another [202].

### 4.1. Amyloid-Beta

**Figure 2:**
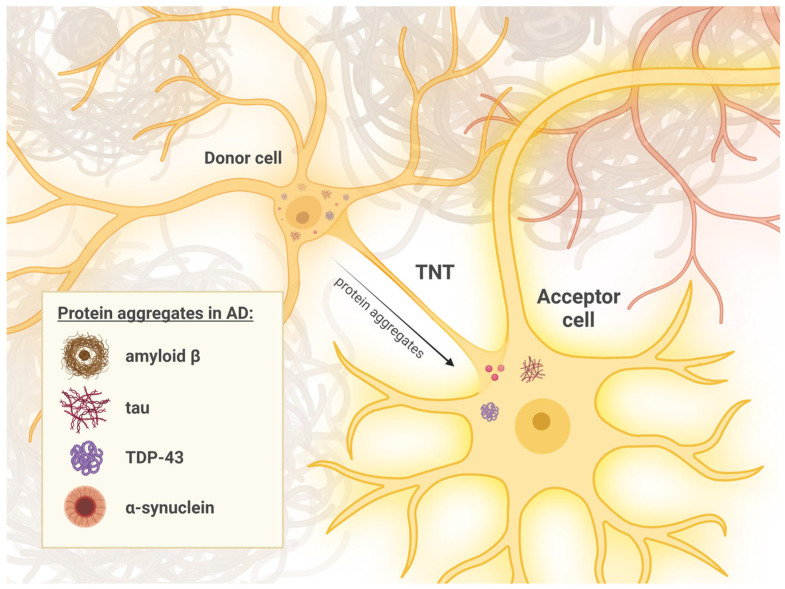
Evidence indicates that amyloid beta (Aβ) can move bidirectionally through tunneling nanotubes (TNTs) in various cell lines. In Alzheimer’s disease (AD), excess Aβ released from cells is rapidly transferred to neighboring cells via TNTs, which accelerates disease progression. Tau may also trigger TNT formation, and fibrillar Tau transport through TNTs has been confirmed in vitro. Both exogenous and endogenous Tau aggregates can be transmitted between cells and have been detected inside TNTs in neuronal cell lines. Alpha-synuclein (α-syn) may be transferred via TNTs in vitro, reducing its burden in donor cells. Similar to Tau, α-syn increases the number of TNT connections compared to untreated cells and can be transported between neurons and microglia. α-Syn aggregates are preferentially transferred from neuronal to microglial cells, while mitochondria are transported in the opposite direction. Additionally, in lymphoblasts from AD patients, increased formation of actin protrusions resembling TNTs or TNT-like structures was observed. Transactive response DNA-binding protein of 43 kDa (TDP-43) aggregates were found alongside F-actin fibers in the cytosolic compartment of these cells and were also detected within tubular actin channels.

There is evidence that Aβ can be transported in both directions within various cell lines through TNTs. An important fact, due to the pathogenesis of AD, is that in pathological conditions, excess Aβ is released from the cells. This release follows the cascade of cell death. The neighboring cells then take up these aggregates and accelerate the progression of AD by quickly and directly transporting the Aβ to other surrounding cells via TNTs [203] (Figure 2). Moreover, in vitro studies demonstrated that oligomeric Aβ triggers plasma membrane (PM) damage and the activation of p21-activated kinase1 (PAK1) remodeling cytoskeletal actin, which results in the formation of elongated F-actin extensions between cells, leading to the creation of membrane conduits resembling TNTs [52]. Considering the above data, which suggest that Aβ not only moves directly between neurons but also induces the formation of pathways for this transport, focusing on TNTs as a target for new drugs is important. This approach could lead to more effective therapies for AD.

Increasing evidence indicates that astrocytes play a significant role in the development of AD. Studies have revealed that although astrocytes effectively consume soluble Aβ aggregates, their ability to degrade them is limited. Consequently, Aβ aggregates build up within astrocytes as intracellular deposits, causing a dysregulation in their physiological function. Moreover, partial breakdown of Aβ may result in the release and dissemination of shortened peptides that pose toxicity to nearby neurons [204]. In AD, astrocytes react to changes in the microenvironment by becoming activated, a phenomenon referred to as astrogliosis. Reactive astrocytes are primarily located in the vicinity of Aβ plaques and are recognized for their elevated production of Aβ, thereby intensifying the progression of AD pathology, by not adequately performing their functions, which include supplying metabolic and trophic support to neurons and assisting in the clearance of proteins [205,206,207]. It has been demonstrated that astrocytes subjected to stress from the presence of Aβ respond by forming TNTs. However, direct transport of Aβ between these cells has not been observed. Instead, a frequent exchange of organelles between astrocytes with Aβ inclusions has been detected, which may indicate dysregulation in the functioning of Aβ-affected astrocytes [204]. It was previously believed that astrocytes in human AD brains are loaded with Aβ42 and act as a storage for this pathological protein due to their limited capacity for degrading the material. However, recent research has demonstrated that human astrocytes, when exposed to Aβ25-35 (an analog of Aβ42), can produce, accumulate, and release Aβ42 themselves. This indicates that astrocytes may play a significant role in AD neuropathology, considering that they surround synapses with their processes, thus contacting numerous neurons [205,208,209].

### 4.2. Tau

In AD, hyperphosphorylated Tau proteins aggregate to form NFTs inside neurons, causing disrupted microtubule networks that impair neuronal transport and synaptic function, and ultimately lead to cell death [210]. Abnormal Tau proteins can spread from neuron to neuron, contributing to the progression of AD. Tau may have the potential to initiate the formation of TNTs. Treatment of mouse CAD (mouse catecholaminergic neuronal cell line) cells with Tau monomers and fibrils resulted in the formation of TNTs in 63% of cells exposed to Tau, compared to only 24% of the untreated cells [54]. Additionally, the neuron-to-neuron transport of fibrillar Tau through these TNTs was confirmed. It has been suggested that during the progression of AD, dead neurons may release phosphorylated Tau and Aβ, which could be subsequently absorbed by the surrounding cells and transmitted via TNTs, significantly accelerating the progression of AD (Figure 2). Bidirectional transport of both Tau protein and Aβ through TNTs has been observed in cell cultures using time-lapse imaging. Interestingly, the uptake rate of Tau protein by TNTs was ten times faster than direct uptake from the culture medium. Similarly, the TNT uptake rate of Aβ was several folds higher [211]. Moreover, Tau aggregates may impede their own degradation through the autophagic pathway and can be trafficked to neighboring cells via TNTs. Both exogenous and endogenous Tau aggregates can be transmitted between cells in a contact-dependent manner and have been detected inside TNTs in neuronal cell lines. These observations confirm that endogenously formed Tau aggregates may propagate via TNTs in a cell contact-dependent mechanism. Co-culturing cells with synthetic Tau fibrils and CK666 (an Arp2/3 inhibitor known to decrease filopodia formation and increase TNT formation by altering the molecular structure of actin regulatory complexes and enhancing filamentous actin polymerization) resulted in a 30% increase in synthetic Tau fibril transfer, while secretion-mediated transfer remained unchanged [194,212,213]. Conversely, sparse cell plating, which inhibits TNT formation due to increased cell distance, significantly decreased the percentage of acceptor cells with synthetic Tau fibrils [55,214]. This suggests that TNT-dependent transfer predominates in these cell models, although mechanisms such as exocytosis and endocytosis could also facilitate fibril transfer between closely situated cells. However, it cannot be ruled out that the full-length Tau spread via secretion modes might be more relevant in vivo compared to the in vitro cell culture model.

TNTs may also serve a protective function against Tau pathology. Recent research has revealed that microglia form TNTs, which connect distant cells, including neighboring microglia. Microglia play a critical role in clearing pathological protein aggregates [215], as they exhibit the highest capacity among all brain cells for scavenging extracellular proteins. Co-culturing neurons with microglia and promoting TNT formation rescues suppressed neuronal activity caused by Tau. Through TNTs, microglia can alleviate neurons from their Tau aggregates and effectively degrade the received protein, which reduces levels of reactive oxygen species in neurons and supports their function and integrity [216].

### 4.3. Other Proteins

It has been shown that non-amyloid components identified as fragments of α-syn are present in Aβ plaques. Furthermore, it has been demonstrated that α-syn oligomers can induce the formation of Aβ oligomers and stabilize their β-cross structures. Additionally, α-syn monomers and fibrils promote Tau aggregation. The interaction between these two proteins also stimulates Tau phosphorylation. In the AD brain, α-syn may increase oxidative stress, lysosomal leakage, and mitochondrial dysfunction by forming hybrid multimers with Aβ and creating nanopore-like structures in the organelles and the plasma membrane. Additionally, elevated α-syn levels have been shown to facilitate Aβ oligomerization as well as activate numerous kinases leading to Tau phosphorylation, and aggregation [217].

Recently it has been reported that α-syn may be transferred via TNTs in in vitro cultures, efficiently reducing the total burden of α-syn in the donor cells. Similarly to the results of Tau treatment, α-syn elevates the total number of intercellular connections in comparison to untreated cells [199,218] (Figure 2). The process of a-syn transfer via TNTs may be crucial for neurodegeneration progression as it can reduce the burden of aggregated α-syn in overloaded microglia and allow α-syn to be degraded by proteinase K in the neighboring naive acceptors [217]. On the other hand, naïve microglia may, in turn, transport their intact mitochondria to the neighboring cells and thereby rescue acceptor cells from cell death via attenuating the intrinsic apoptotic signaling pathway. Importantly, the formation of TNTs between microglia was demonstrated in organotypic slice cultures, in vivo in mice, as well as in the immunostainings of PD and MSA patients’ brain sections [54]. TNTs may also mediate the transport of a-syn between neurons and microglia. A recent study has shown that, in vitro, α-syn aggregates can be transferred with a high degree of bias from neuronal to microglial cells, while mitochondria are preferentially transported in the opposite direction [202]. Interestingly, TNTs were also shown to mediate the inter-cellular transport of other organelles, namely lysosomes [219].

Additionally, similar to Aβ, it has been shown that under oxidative stress, neurons containing aggregated pathological deposits of α-syn form TNTs [201]. The increased presence of TNTs would facilitate the transmission of aggregates between cells, thereby aiding in the propagation of pathogenic clusters. Understanding the process of TNT formation induced by aggregates is a critical area of research, essential for unraveling the pathways and molecules involved in this phenomenon.

Moreover, a correlation between TDP-43 protein and AD has been suggested, given the frequent observation of TDP-43 pathology in post-mortem AD brains, which has been associated with severe brain atrophy and increased memory impairment [220]. TDP-43, a widely distributed protein expressed in nearly all human tissues, belongs to the heterogeneous nuclear ribonucleoprotein (hnRNP) family and is primarily localized in the nucleus, where it regulates RNA transcriptional control, alternative splicing, and mRNA stabilization [221,222]. However, under pathological conditions, TDP-43 can undergo cleavage, hyperphosphorylation, and ubiquitination. As a result of these posttranslational modifications, TDP-43 may accumulate, and form aggregates localized in the cytoplasm, often co-localizing with NFTs and senile plaques [223].

TDP-43 could be involved in several pathological mechanisms associated with AD including the following: Aβ accumulation, Tau hyperphosphorylation, mitochondrial dysfunction, and neuroinflammation [224,225,226]. Numerous experiments have been carried out to demonstrate the cell-to-cell propagation of TDP-43 in a cell culture setting [227,228,229]. It has been demonstrated that, both in vitro and in vivo, the aggregation and transmission of TDP-43 could be controlled by the exosome pathway. However, another potential mechanism for the propagation of TDP-43 protein involves TNTs. In a study conducted by Cuevas et al., the distribution of TDP-43 aggregates in the lymphoblasts of AD patients was examined using immunofluorescence staining [230]. An investigation of F-actin revealed cytoskeletal abnormalities, including an increased formation of actin protrusions resembling TNTs or TNT-like structures. These structures were much more pronounced in AD patient cells than in control cells, and their quantity correlated with the severity of the clinical presentation. TDP-43 aggregates were observed alongside F-actin fibers in the cell’s cytosolic compartment, with TDP-43 aggregates additionally detected within tubular actin channels [177].

Furthermore, several studies have revealed that overexpression of TDP-43 in astrocytes can induce non-cell autonomous neuronal toxicity [231,232]—a condition where damage or toxicity affects neurons without originating directly from the neurons themselves. Instead, it typically arises from neighboring cells or surrounding tissues, such as glial cells (like astrocytes) or other non-neuronal cells. In the context of neurodegenerative diseases, this concept suggests that pathological processes initiated in non-neuronal cells can influence neuronal health and function, contributing to disease progression. Therefore, the propagation of TDP-43 by glial cells may also play a significant role in the progression of neurodegenerative diseases [231,232,233,234] (Figure 2).

## 5. Conclusions

Over the years, multiple hypotheses regarding the etiopathogenesis and progression mechanisms of AD have been investigated. Although the Aβ and Tau hypotheses are well-studied and established, researchers have recently begun to analyze the role of α-syn and TDP-43 protein, as well as microglia and astrocytes in the development of the disease. Moreover, the current investigations into the spread of AD pathology within the brain have increasingly focused on the formation and functions of TNTs. Numerous in vitro studies have confirmed the possibility of transferring pathological proteins between CNS cells via TNTs, but more in vivo studies are needed to elucidate this phenomenon fully and precisely.

Intriguingly, TNTs may play a dual role in AD by not only facilitating disease progression but also exerting neuroprotective effects. Through TNTs, neurons can transfer α-syn to glial cells while concurrently acquiring healthy mitochondria, thereby mitigating cellular stress associated with the accumulation of pathological proteins. This potential for TNTs to alleviate the neuronal burden of α-syn—one of the key pathological proteins implicated in AD—suggests that TNTs could be explored as a therapeutic avenue in the treatment of AD.

Despite the established roles of TNTs in various pathological contexts, including oncogenesis, infectious diseases, and other neurodegenerative disorders, their potential application in AD remains unexplored in clinical conditions. TNTs represent a nascent and evolving field of study that warrants further in-depth investigation. The inherent fragility of TNTs, coupled with their susceptibility to a range of external and internal stimuli, poses significant challenges for their study. Additionally, the absence of a universal, specific marker for TNTs further complicates their characterization and analysis. Given the limited efficacy of current Aβ-targeted therapies and the persistent lack of pharmacological interventions capable of effectively arresting neurodegeneration in AD, continued research into TNTs is imperative. Advancing our understanding of TNTs could provide critical insights into AD pathogenesis and may pave the way for the development of innovative therapeutic strategies aimed at mitigating or halting the progression of this devastating disease.

## Figures and Tables

**Table 1:** A table summarizing the role of two types of TNTs in the mechanism of AD pathology.

Type of TNTs	Description	Mechanism of AD Pathology
“thin” TNTs	Cell junctions made of actin with a maximum diameter of 700 nm. They can only transport small molecules with a maximum mass of 1.2 kDa.	Not fully known.
“thick” TNTs	Cell junctions made of actin and microtubules with a diameter exceeding 700 nm. They are significantly more stable and can transport large molecules with a mass greater than 1.2 kDa, organelles, and viruses.	TNTs facilitates the transport of molecules such as tau, α-syn, and Aβ to spread neuronal damage.

## Data Availability

The data generated in the present study may be requested from the corresponding author.
